# Evaluation of a hybrid pipeline for automated segmentation of solid lesions based on mathematical algorithms and deep learning

**DOI:** 10.1038/s41598-022-18173-0

**Published:** 2022-08-20

**Authors:** Liam Burrows, Ke Chen, Weihong Guo, Martin Hossack, Richard G. McWilliams, Francesco Torella

**Affiliations:** 1 Centre for Mathematical Imaging Techniques and Department of Mathematical Sciences, University of Liverpool, Liverpool, L69 7ZL UK; 2 Department of Mathematics, Applied Mathematics and Statistics, Case Western Reserve University, Cleveland, OH 44106 USA; 3 Department of Radiology, Liverpool University Hospitals, Liverpool, UK; 4 Liverpool Vascular and Endovascular Service, Liverpool University Hospitals NHS Foundation Trust, Liverpool, UK

**Keywords:** Mathematics and computing, Medical imaging

## Abstract

We evaluate the accuracy of an original hybrid segmentation pipeline, combining variational and deep learning methods, in the segmentation of CT scans of stented aortic aneurysms, abdominal organs and brain lesions. The hybrid pipeline is trained on 50 aortic CT scans and tested on 10. Additionally, we trained and tested the hybrid pipeline on publicly available datasets of CT scans of abdominal organs and MR scans of brain tumours. We tested the accuracy of the hybrid pipeline against a gold standard (manual segmentation) and compared its performance to that of a standard automated segmentation method with commonly used metrics, including the DICE and JACCARD and volumetric similarity (VS) coefficients, and the Hausdorff Distance (HD). Results. The hybrid pipeline produced very accurate segmentations of the aorta, with mean DICE, JACCARD and VS coefficients of: 0.909, 0.837 and 0.972 in thrombus segmentation and 0.937, 0.884 and 0.970 for stent and lumen segmentation. It consistently outperformed the standard automated method. Similar results were observed when the hybrid pipeline was trained and tested on publicly available datasets, with mean DICE scores of: 0.832 on brain tumour segmentation, and 0.894/0.841/0.853/0.847/0.941 on left kidney/right kidney/spleen/aorta/liver organ segmentation.

## Introduction

Volumetric assessment of solid lesions has been revolutionised by modern cross-sectional imaging. Presently, such assessment is performed by radiologists using manual or semi-automated segmentation tools provided by various software packages. This process can be time-consuming, particularly when comparative analysis of serial scans is required. Fully automated segmentation could, potentially, greatly accelerate this process and improve its accuracy by removing the bias associated with operator dependency. Recent work into image segmentation has focused on two main types: model-driven^1–3^ and data-driven methods^4–6^. Model-driven methods utilise mathematical (variational) formulations, which have been shown to provide a powerful framework for segmenting images. A variational method for segmentation can be composed of a number of terms, each hand crafted to achieve a desired result in the output; essentially image features desirable to a particular segmentation task can be built into the model (for example a desired segmented object may be found using a combination of a particular image intensity, shape, and/or location in the image), and the resultant algorithm is run independently on each image (for example: a computed tomography slice).

Data-driven methods have drawn attention in recent years due to the rise of deep learning and powerful computing hardware. Segmentation methods using deep learning methods such as convolutional neural networks (CNNs), are considered the gold standard, if provided with enough data.

One challenge for deep learning segmentation is the acquisition of an appropriately large dataset with ground truth labels, which can be both an expensive and time-consuming process. Ground truth labels are usually manual segmentations of the region of interest performed by an expert. They are necessary for training deep learning algorithms and for evaluating their performance. To address this problem, hybrid approaches to segmentation have been proposed, whereby variational methods are used to supplement manual ground truth labels^7–9^, thus reducing the demand for manual hand drawn segmentation.

We have developed our own fully automated hybrid approach (a hybrid segmentation pipeline) for volumetric segmentation, which combines a recently developed variational model^10^ with a deep learning algorithm. In this work, we tested the accuracy of this hybrid segmentation pipeline.

## Methods

### Reference standards

To test our pipeline, we chose contrast-enhanced computed tomography (CT) angiograms of patients being followed up after endovascular repair (EVAR) of abdominal aortic aneurysms (AAAs), as the stented aorta is an example of a solid lesion that requires serial follow-up with cross-sectional imaging. We chose post-EVAR CTs because serial comparison of scans is a common diagnostic problem, which can be time-consuming and labour intensive. The choice was also due, in part, with familiarity (three authors are vascular interventionists), and to the fact that our group had previously used and tested a reproducible manual segmentation technique on these scans^11,12^. CTs were performed with a 64-slice Siemens Somatom scanner (Siemens Healthcare, Frimley, UK). Manual segmentation was performed by one of the authors (MH) on reconstructed 2 mm thick slices with intervals of 2 mm, according to a previously described technique^11–13^.

We acquired a total of 70 fully anonymised postoperative CTs, of which 50 were manually segmented to provide a “ground truth” for training the deep learning algorithm, and to provide useful evaluation metrics. The manual segmentation (“ground truth labelling”) was conducted between the lowermost renal artery and the aortic bifurcation, by hand, using an open source application called ITK-SNAP^14^. This segmentation was considered the reference standard.

We used a typical 60:20:20 ratio for training, validation, and testing the images: 30 sets to train the deep learning part, 10 sets to validate and prevent overfitting, and reserved the final 10 sets for evaluation purposes. Our pipeline also used 20 unlabelled datasets during the training phase, providing us with 50 volumes in total during training.

Additionally, we evaluated the pipeline on two publicly available datasets: The Brain Tumour Segmentation challenge (BraTS)^15–17^, and the Abdomen data from the Multi-Atlas Labelling Beyond the Cranial Vault challenge^18^. The BraTS dataset contains a range of MR modalities, but for our purposes we considered only the fluid attenuated inversion recovery (FLAIR) volumes. All sets have been labelled by one to four raters following the same protocol, and their annotations approved by experienced neuro-radiologists. We segmented only the tumour region in each volume. We used a total of 200 volumes for the BraTS data: 120 during training, 40 for validation and 40 for evaluation purposes. In addition to the BraTS, we used the Abdomen dataset, a collection of CT scans of the abdomen in which 13 organs have been segmented by two experienced undergraduate students and the segmentation verified by a radiologist. We evaluated our pipeline on five of the 13 organs: the spleen, the right kidney, the left kidney, the liver and the aorta. The Abdomen dataset contains 30 scans; of these, we used 15 for training, 5 for validation and 10 for evaluation.

### Pipeline tests

After windowing and selecting the uppermost and lowermost slice for segmentation on either a post-EVAR aneurysm or a selected organ from the BraTS or Abdomen datasets, we ran our variational model^10^. This original model uses an enhanced method of edge detection, which allows for images containing low contrast to be segmented effectively. Following edge detection, the region of interest is segmented based on image intensity and pixel location in the image^10^. The variational method provided us with a good but not perfect initial segmentation, as some regions may contain no contrast at the boundary. Furthermore, artifacts may result in poor definition in certain areas.

Although it is possible to obtain accurate segmentation by using the variational method only, this is a time-consuming process, taking up to 20 min for a large volume. Further, each new volume would require a user to manually insert a set of markers to indicate the region of interest. We eliminated this step entirely by using image registration. In practice, we ran the variational segmentation model for one 3D volume (aneurysm, tumour or organ), obtaining an initial segmentation. For each subsequent scan we simply superimposed this segmentation to the new scan, thereby registering the saved segmentation onto the new image, rather than re-running the variational method on each new image. The overlapped images were then registered by a previously trained network^19^, which, although not entirely accurate, produced an estimate that could be fed to the CNN, to produce an accurate final result. This image with the initial segmentation was not excluded, and maintained a place in the training set. This registration step removed the need for user interaction, making the method automatic.

The final stage involved feeding each estimated segmented volume to the CNN. Unlike in commonly used methods of segmentations by CNNs^4,6^, ours received both the scan and the estimated segmented volume (the output of the variational model). This provided the CNN with supplementary information to produce an accurate final result. The CNN was trained in a standard way using backpropagation (see appendix for details).

“Unlabelled” scans (i.e., image volumes without manual segmentation) were also used to train our pipeline. The estimated volume from the variational method was used here in place of the reference standard. This allowed us to expand our training set, exposing the network to more data without needing more time-consuming manual labeling. This is commonly known as a semi-supervised approach to learning (see appendix).

### Data analysis and presentation

The accuracy of an automated segmentation pipeline depends on its ability to correctly identify all voxels, hence volume, belonging to an organ/lesion in a scan, as well as those that lie outside said organ/lesion. To evaluate the accuracy of our pipeline against manual segmentation we reported true positives (TP) as the number of voxels correctly identified by a segmentation method; false positives (FP) and false negatives (FN), as the number of voxels incorrectly identified/excluded by a segmentation method; sensitivity as the ability of a model to correctly identify all relevant voxels or volume, and false negative rate (the inverse of sensitivity). We did not report true negatives, as these are largely dependent on the total number of voxels included in a scan (usually a whole section of the body), hence values are always very high. Continuous variables were expressed as median and range, as they were generally not normally distributed. Correlation was evaluated by graphical methods and agreement with Bland–Altman plots^20^.

We also used more commonly used segmentation metrics to evaluate the pipeline, including the DICE, the JACCARD and the volumetric similarity (VS) coefficients, which are defined as:

$$\begin{aligned} DICE = \frac{2 TP}{2 TP + FP + FN}, \quad JACCARD = \frac{TP}{TP + FP+ FN}, \quad VS = 1 - \frac{ |FN - FP| }{ 2 TP + FP + FN}. \end{aligned}$$

Finally, the Hausdorff Distance (HD) between a segmented volume X and a ground truth segmentation GT is defined as:

$$\begin{aligned} HD(X,GT) = \max \big ( h(X,GT), h(GT,X) \big ), \end{aligned}$$

where *h*(*X*, *GT*) is the directed Hausdorff distance given by:

$$\begin{aligned} h(X,GT) = \max _{x \in X} \min _{y \in GT} | x - y |, \end{aligned}$$

where $|x-y|$ is the Euclidean distance between two points, where *x* is in *X* and *y* is in *GT*.

Both DICE and JACCARD scores range between 0 (where no overlap between output and reference occurs) and 1 (where the output is exactly the reference segmentation). VS represents how similar the volume of the segmented output is to the volume of the reference segmentation. This is not influenced by the overlap: two organs with the exact same volume but in different positions would result in a VS score of 1. Finally, HD describes the largest distance from one volume to the nearest point in the other. Unlike the other metrics described here, the ideal result of HD is a score of 0, as the voxels of an ideal segmentation would be in the same place as those of the reference standard.

In order to compare our pipeline to a more traditional method of automated segmentation, we trained a deep learning model in a typical fashion (using only the scan as input, without the input of our variational model). This method is referred as “standard” in our results.

As further experiments, we trained the hybrid method with a decreasing number of data in the training set in order to determine if the hybrid approach can perform better than the standard method when provided with less data. In addition we run the hybrid method on some simplified networks with lighter architectures (see appendix).

### Ethical approval

The study was conducted in accordance with relevant institutional guidelines and regulations.

## Results and discussion

### Results

#### Aortas

**Figure 1:**
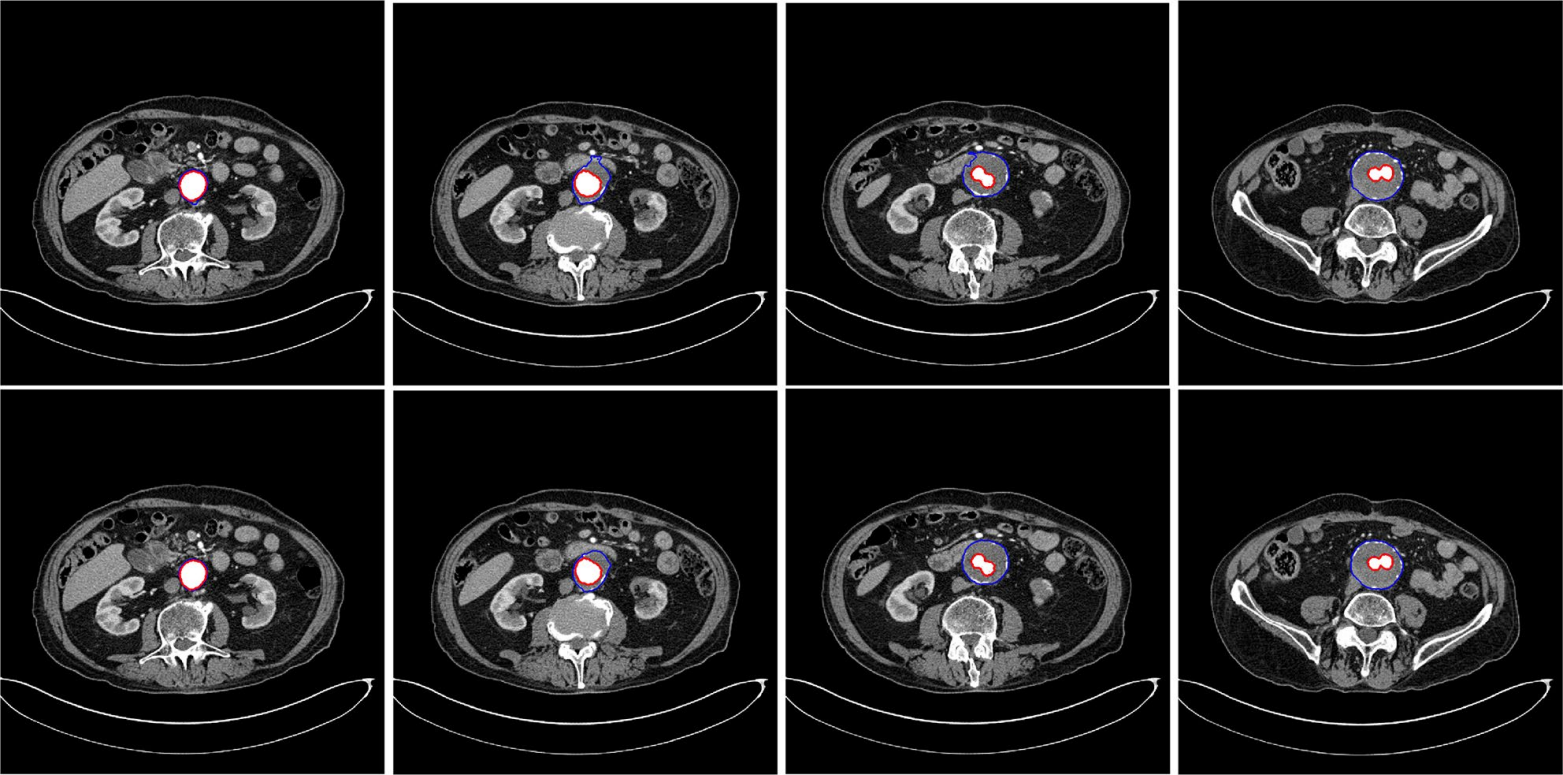
An example of segmentation by the two pipelines, with axial cross-sections of an aneurysm at four different levels. The standard pipeline is shown on the top row, with the hybrid pipeline on the bottom row. Here the standard pipeline is unable to accurately follow the contour of the aneurysm. The hybrid pipeline appears accurate.

**Figure 2:**
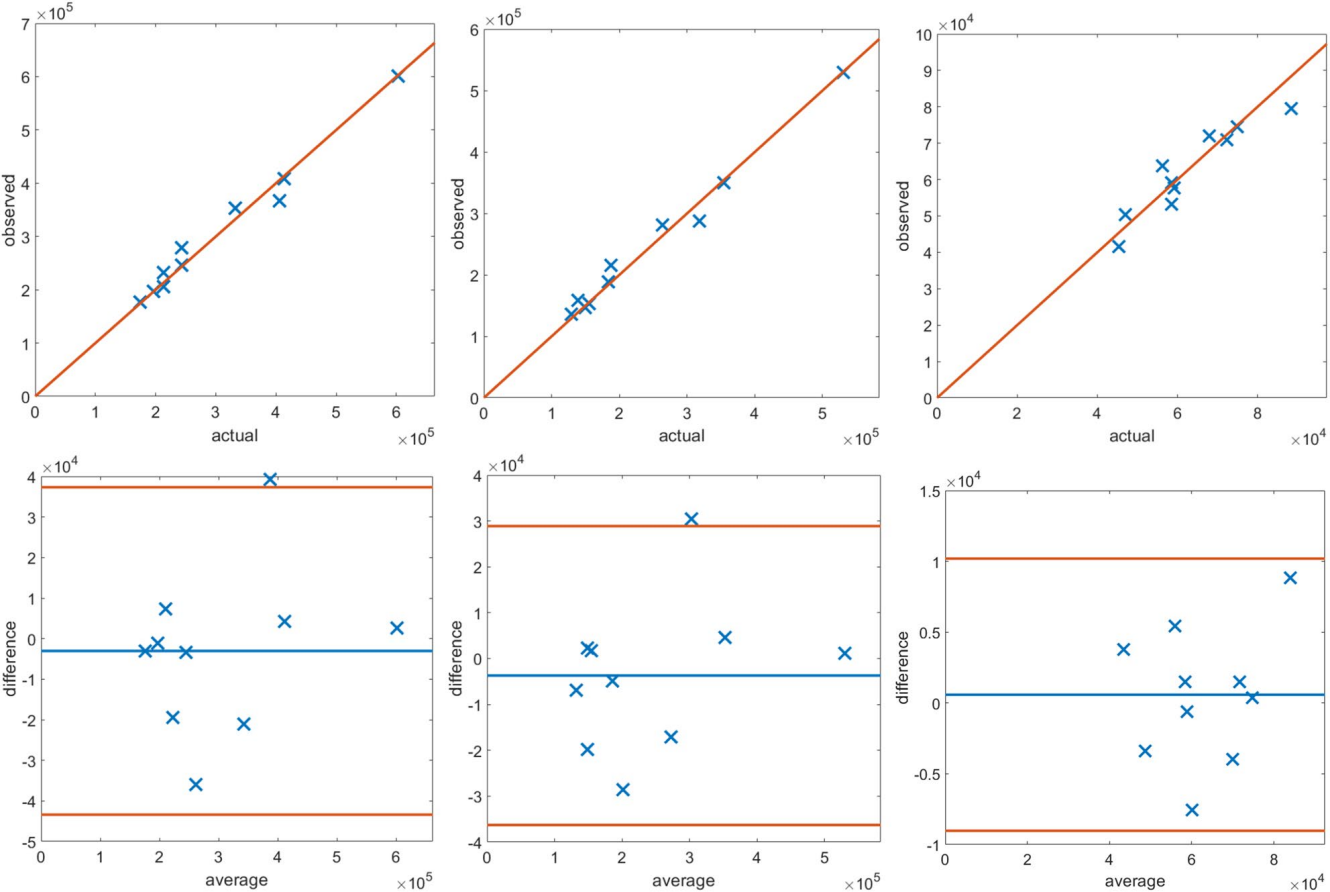
Correlation (top row) and agreement (bottom row) between the proposed hybrid approach and the ground truth in voxel detection for the aortic data. Units are the number of voxels.

Figure 1 displays a 2D example output of both approaches, showing various cross-sections of a 3D volume. On manual segmentation, the median (range) thrombus volume was 185 (129–531) ml, corresponding to 179,303 (111,336–588,375) voxels, whereas the stent and lumen volume was 59 (45–88) ml, corresponding to 55,717 (36,117–102,904) voxels. The accuracy of the “standard” and hybrid pipelines is reported in Tables 1 and 2. Notably, the hybrid method had slightly more TPs, less FPs and more FNs, resulting in higher DICE, JACCARD and VS for the whole aneurysm and the thrombus. Correlation and agreement between the measurements of the two pipelines is displayed in Fig. 2.

#### Abdomen/BraTS

Results of the segmentation of abdominal organs are displayed in Tables 5 and 6. For all organs, the hybrid pipeline was more accurate than the standard, producing more true positives and fewer false positives or negatives than the standard one, except for the spleen, in whose segmentation the hybrid pipeline resulted in a higher median number of false negatives. Similarly, the hybrid pipeline also outperformed the standard one in the segmentation of brain tumours (Tables 3, 4).

#### Further experiments

Results from further experiments can be found in the appendix. Of particular note was the experiment which trained networks using the hybrid approach with the three databases, but with reduced data. Mean DICE values for the thrombus/stent and lumen/whole aneurysm were 0.877/0.927/0.903 when 20 supervised volumes were in the training data (instead of 30 in the original experiments), outperforming the standard method with full data. Similar trends were seen with the BraTS dataset with only 60 volumes (instead of 120) giving mean DICE values of 0.761 and in the Abdomen dataset trained with 10 (instead of 20) giving mean DICE values of 0.867/0.807/0.841/0.823/0.922 for left kidney/right kidney/spleen/aorta/liver segmentation.

### Discussion

Our results suggest that the combination of a mathematical (variational) approach with a deep learning algorithm may improve the accuracy of automated segmentation of organs and tumours on CT.

For general purposes, variational and deep learning segmentation have been studied widely, though limited work has been conducted into hybrid approaches^7–9^. Recently, deep learning has become the preferred approach to segmentation and can produce outstanding results on a wide variety of applications^21–23^. Wang et al.^24,25^ used a similar approach to ours, albeit fully based on deep learning. In their proposed method, an initial network produces an initial segmentation; if this is not desirable a second network is used to refine the result. Our proposed method is similar, but, in place of a network, the first step is a variational algorithm, which we recently developed specifically to improve segmentation in the presence of low contrast^10^.

Use of the variational method is innovative, and it allows us greater control over the initial segmentation when compared with the work by Wang et al.^24,25^. Further, a major weakness of deep learning methods is their heavy reliance on large, labelled datasets. As we can rely on the variational method to provide a reasonably good initial segmentation, we can also use unlabelled data in the training stage, thus expanding our training dataset. The output segmentation should therefore be more resilient to variation.

#### Aortic segmentation

Automated segmentation of the abdominal aorta has been addressed before to some success. Early approaches include variational model-based methods such as level set methods (a mathematical way of representing a shape) by Loncaric et al.^26^ and Subasic et al.^27,28^, whereas Zohios et al.^29^ used level sets and geometrical methods to segment the thrombus in the presence of calcifications. These models can be very time-consuming, and susceptible to imprecision where low contrast is present.

More recently Lalys et al.^30^ proposed a fast 3D model based on the snakes model by Kass et al.^31^, capable of segmenting both the lumen and thrombus but requiring some user input. While quoting a mean DICE score of 0.87 on post operative CTA scans, use of a shape based deformable model, using image registration to achieve segmentation, can suffer on unusual scans. Based on a similar idea, Lareyre et al.^32^ proposed a fully automated pipeline for segmentation of AAAs, obtaining both lumen and thrombus incorporating the Chan-Vese model^2^. Evaluation of thrombus segmentation was performed on 525 selected slices from 40 CT scans, giving a mean: DICE of 0.88, Jaccard of 0.80 and Sensitivity of 0.91, which performs slightly worse than our pipeline’s mean results of 0.91, 0.84 and 0.92 respectively.

Deep learning methods include that by Lopez et al.^33^, who developed a fully automatic approach for segmenting the thrombus on CT scans of patients treated with Endovascular Aneurysm Repair (EVAR) using a new network architecture based on the proposed work by Xie et al.^34^. In a follow up work^35^, the authors extended their work to segment 3D volumes, maintaining the fully automatic aspect. Notably, segmentation was performed both on preoperative and postoperative CT scans, with a mean DICE score of 0.89 and Jaccard of 0.81 on segmentation of the whole aneurysm on postoperative scans. Lu et al.^36^ proposed a 3D pipeline using a V-Net architecture^37^ combined with an ellipse fitting to estimate the maximum diameter of the aorta. Both contrast and non-contrast enhanced scans were used, quoting DICE scores of 0.89 and 0.90 on preoperative scans with and without contrast respectively. More recently the work by Caradu et al.^38^ proposed a deep learning algorithm trained to segment preoperative infrarenal aortic aneurysm CT volumes effectively, with a mean DICE score of 0.95 on 100 scans. Adam et al.^39^introduced an automated method named Augmented Radiology for Vascular Aneurysm (ARVA), trained on a large dataset of 489 CT volumes (a combination of both preoperative and postoperative scans), dedicated to segmenting the entire aorta from the ascending portion to the iliac arteries, with a mean DICE score of 0.95 on preoperative scans and 0.93 on postoperative scans, thus comparable to ours, but achieved with fewer training data. This study nevertheless confirms that the use of an initial variational method can reduce the need for larger datasets.

#### BraTS

The BraTS dataset is a widely used imaging dataset in the literature^40–42^. All scans include several MR sequences, including T1, post-contrast T1-weighted, T2-weighted and T2-FLAIR. Labels are also provided for the whole tumour, tumour core, and enhancing tumour regions. In order to simplify the experiments, we only segmented the T2-FLAIR sequence, which is commonly used in brain imaging. The BraTS dataset draws a fair amount of attention: for example, Jiang et al.^40^ developed a two-stage model using a U-Net^4^, utilising all sequences and using a post-processing thresholding technique. If the enhancing tumour region was less than a hand-tuned threshold, then the region was replaced with necrosis, which may cause significant improvement to the results . An average DICE of 0.888 for tumours was reported. Zhao et al.^41^ made use of a pipeline involving a CNN and a number of expedients including different methods of sampling, patch-based training and teacher-student models, resulting in a reported mean DICE score of 0.883. Ali et al.^42^ exploited multiple CNNs trained separately, with final predictions based on ensembling the probability maps from each CNN, with a mean DICE of 0.906 for the whole tumour. These works developed specific pipelines with a particular focus on brain tumour segmentation, making use of all available sequences. Our proposed pipeline, which was not brain-specific, did not produce quite as good results but was only based on T2 FLAIR sequences. It is possible that its accuracy would have been greater if more sequences had been used. It is also possible that its performance on MR-acquired images and/or brain images may not as good as that on CT-acquired images and/or aortic ones.

#### Abdominal organs

Gibson et al.^43^ proposed a new network architecture for the purpose of multi-organ segmentation in abdominal CT scans. A mean DICE of 0.93, 0.95, 0.95 was reported for the left kidney, the spleen and liver respectively. Another method was developed by Cai et al.^44^ who developed a novel shape learning network architecture, building an expected shape for the organ into the model. A mean DICE score of 0.96 and 0.94 was reported for the spleen and liver respectively. Weston et al.^45^ implemented a deep learning method aimed at segmenting the complete abdomen and pelvis using a locally collected dataset. A variation on the 3D UNet was implemented, and a mean DICE score of 0.93, 0.93, 0.88, 0.95 was reported for the kidneys(combined), spleen, aorta and liver respectively. These methods were developed specifically for abdominal segmentation. Although our pipeline was not quite as accurate, the quantitative results demonstrate the advantage of the hybrid pipeline over a conventional approach.

A summary of the discussed methods from the literature and the proposed method can be found in Table 7.

The main limitation of our study is the limited testing of the hybrid pipeline. It is possible that its accuracy may not be reproducible when applied to other organs, to scans performed with different settings (for example: inferior resolution), to patients with less well-defined boundary conditions between body structures on CT or MR, or in presence of artefacts. Notably, all the above issues would also interfere with manual segmentation, which is still considered the gold standard. More extensive training/testing of the pipeline would be necessary to clarify its generalisability.

## Conclusion

Our fully automatic segmentation pipeline combining elements from both mathematical modelling and artificial intelligence has been shown to be an accurate method for segmenting aortic 3D volumes and performed well also when applied to MR images of brain tumours and abdominal organs.

We plan to test and refine the pipeline further on similar but not identical clinical tasks, such as segmentation of preoperative CT scans of the aorta. This could be achieved by using the current pipeline as a baseline and potentially modifying it to suit preoperative scans. Our segmentation pipeline provides the groundwork for developing a method for serial comparison of images, potentially reducing operator time and bias.

Ultimately, further testing on larger datasets will be necessary before attempting clinical experimentation and translation.

**Table 1:** Standard and Hybrid pipeline accuracy in aneurysm segmentation.

Thrombus:	Standard	Hybrid
As measured	180,651 (138,491–590,159)	202,160 (135,817–529,732)
TP	160,358 (120,456–493,880)	174,384 (119,911–503,438)
FN	11,708 (3631–167,718)	13974 (4484–56,037)
FP	21,378 (7768–96,279)	19994 (9306–46,248)
**Stent and lumen**		
As measured	60,190 (45,333–74,340)	61,499 (41,540–79,582)
TP	56,863 (42,893–72,441)	56,187 (40,472–74,887)
FN	2074 (220–39,972)	2726 (1504–13,532)
FP	2868 (891–8112)	2680 (1045–9293)
**Whole aneurysm**		
As measured	247,781 (183,816–662,998)	262,863 (177,255–600,584)
TP	220,060 (167,745–566,548)	233,612 (167,327–575,127)
FN	9329 (2283–206,380)	13,762 (4582–66,201)
FP	20,447 (7345–96,450)	17,912 (7500–50,589)

Units displayed are volume (mm$^3$), given as median (range).

**Table 2:** Performance on segmentation metrics of the variational method (VM) only, and standard and hybrid pipeline in aneurysm segmentation.

Thrombus	VM	Standard	Hybrid
DICE	0.734 ${\pm }$ 0.088	0.873 ${\pm }$ 0.094	0.909 ${\pm }$ 0.054
JACCARD	0.587 ${\pm }$ 0.107	0.784 ${\pm }$ 0.132	0.837 ${\pm }$ 0.087
VS	0.866 ${\pm }$ 0.117	0.927 ${\pm }$ 0.095	0.972 ${\pm }$ 0.026
HD	41.0 ${\pm }$ 11.4	95.7 ${\pm }$ 81.5	60.4 ${\pm }$ 67.1
TPR	0.660 ${\pm }$ 0.104	0.893 ${\pm }$ 0.150	0.922 ${\pm }$ 0.048
TNR	0.999 ${\pm }$ 0.001	0.999 ${\pm }$ 0.0001	0.999 ${\pm }$ 0.0001
Time (s)	345 ${\pm }$ 31.5	11.6 ${\pm }$ 0.258	11.6 ${\pm }$ 0.258
**Stent and lumen**			
DICE	0.923 ${\pm }$ 0.025	0.928 ${\pm }$ 0.082	0.937 ${\pm }$ 0.028
JACCARD	0.857 ${\pm }$ 0.044	0.875 ${\pm }$ 0.123	0.884 ${\pm }$ 0.045
VS	0.982 ${\pm }$ 0.012	0.955 ${\pm }$ 0.086	0.970 ${\pm }$ 0.022
HD	56.6 ${\pm }$ 68.2	60.7 ${\pm }$ 69.9	49.0 ${\pm }$ 60.4
TPR	0.942 ${\pm }$ 0.027	0.928 ${\pm }$ 0.134	0.935 ${\pm }$ 0.044
TNR	0.999 ${\pm }$ 0.0001	0.999 ${\pm }$ 0.0001	0.999 ${\pm }$ 0.0001
Time (s)	332 ${\pm }$ 38.7	11.6 ${\pm }$ 0.258	11.6 ${\pm }$ 0.258
**Whole aneurysm**			
DICE	0.803 ${\pm }$ 0.058	0.902 ${\pm }$ 0.093	0.933 ${\pm }$ 0.041
JACCARD	0.674 ${\pm }$ 0.079	0.831 ${\pm }$ 0.133	0.877 ${\pm }$ 0.070
VS	0.903 ${\pm }$ 0.081	0.935 ${\pm }$ 0.092	0.976 ${\pm }$ 0.024
HD	55.8 ${\pm }$ 52.4	109 ${\pm }$ 80.0	52.4 ${\pm }$ 55.5
TPR	0.740 ${\pm }$ 0.075	0.918 ${\pm }$ 0.151	0.942 ${\pm }$ 0.043
TNR	0.999 ${\pm }$ 0.001	0.999 ${\pm }$ 0.0001	0.999 ${\pm }$ 0.0001
Time (s)	678 ${\pm }$ 58.5	11.6 ${\pm }$ 0.258	11.6 ${\pm }$ 0.258

Units given as mean ${\pm }$ standard deviation.

**Table 3:** Standard and Hybrid pipeline accuracy in brain tumour segmentation.

BraTS:	Standard	Hybrid
As measured	133,209 (2775–915,633)	95,233 (7253–315,035)
TP	82,413 (2775–215,828)	81,508 (7249–214,419)
FN	5480 (15–80,514)	5047 (594–57,515)
FP	14,077 (0–783,250)	8625 (5–231,803)

Units displayed are volume (mm$^3$), given as median (range).

**Table 4:** Performance on segmentation metrics of the variational method (VM) only, and standard and hybrid pipeline in brain tumour segmentation.

BraTS:	VM	Standard	Hybrid
DICE	0.746 ${\pm }$ 0.148	0.596 ${\pm }$ 0.308	0.832 ${\pm }$ 0.130
JACCARD	0.614 ${\pm }$ 0.159	0.490 ${\pm }$ 0.314	0.730 ${\pm }$ 0.170
VS	0.792 ${\pm }$ 0.156	0.633 ${\pm }$ 0.316	0.863 ${\pm }$ 0.132
HD	28.3 ${\pm }$ 18.0	49.4 ${\pm }$ 29.5	36.8 ${\pm }$ 20.5
TPR	0.637 ${\pm }$ 0.165	0.800 ${\pm }$ 0.262	0.884 ${\pm }$ 0.141
TNR	0.999 ${\pm }$ 0.001	0.998 ${\pm }$ 0.001	0.999 ${\pm }$ 0.001
Time (s)	46.6 ${\pm }$ 6.76	3.71 ${\pm }$ 0.102	3.73 ${\pm }$ 0.123

Units given as mean ${\pm }$ standard deviation.

**Table 5:** Standard and hybrid pipeline accuracy in organ segmentation.

Left kidney:	Standard	Hybrid
As measured	155,878 (70,294–222,822)	158,221 (107,374–220,349)
TP	131,585 (66,150–191,654)	123,870 (101,112–198,947)
FN	11,081 (965–52,481)	7518 (736–55,575)
FP	19,458 (4144–54,788)	15,512 (6262–46,373)
**Right kidney**		
As measured	197,531 (14,369–236,772)	164,799 (79,980–222,058)
TP	132,750 (0–184,937)	132,802 (69,714–188,599)
FN	17,956 (1781–124,803)	14,531 (2098–59,380)
FP	41,861 (4039–99,438)	22,718 (4145–45,210)
**Spleen**		
As measured	185,886 (100,546–352,992)	256,998 (94,334-1,001,976)
TP	171,358 (87,343–315,736)	177,268 (82,381–397,355)
FN	7786 (1176–159,828)	9748 (5203–98,193)
FP	20,012 (3848–43,048)	14,703 (5526–876,251)
**Aorta**		
As measured	81,256 (38,618–200,230)	86,442 (33,559–216,247)
TP	70,117 (12,504–148,501)	64,838 (28,381–197,198)
FN	17,419 (4046–121,927)	9859 (1057–73,310)
FP	15,885 (2880–51,729)	7722 (2407–37,472)
**Liver**		
As measured	1,697,921 (1,329,048–2,333,988)	1,654,217 (1,288,193–2,237,004)
TP	1,601,916 (1,024,422–2,182,113)	1,602,463 (1,025,609–2,150,051)
FN	78,860 (28,886–202,664)	65,631 (27,699–281,215)
FP	121,130 (39,374–314,559)	86,373 (40,837–262,584)

Units displayed are volume (mm$^3$), given as median (range).

**Table 6:** Performance on segmentation metrics of the Variational Method (VM) only, and Standard and Hybrid pipeline in organ segmentation. Units given as mean ${\pm }$ standard deviation.

Left kidney:	VM	Standard	Hybrid
DICE	0.872 ${\pm }$ 0.021	0.856 ${\pm }$ 0.072	0.894 ${\pm }$ 0.077
JACCARD	0.773 ${\pm }$ 0.033	0.753 ${\pm }$ 0.104	0.815 ${\pm }$ 0.114
VS	0.886 ${\pm }$ 0.033	0.904 ${\pm }$ 0.074	0.954 ${\pm }$ 0.041
HD	5.89 ${\pm }$ 6.31	9.70 ${\pm }$ 3.04	7.47 ${\pm }$ 8.52
TPR	0.784 ${\pm }$ 0.042	0.867 ${\pm }$ 0.149	0.904 ${\pm }$ 0.114
TNR	0.999 ${\pm }$ 0.0001	0.999 ${\pm }$ 0.0001	0.999 ${\pm }$ 0.0001
Time (s)	26.7 ${\pm }$ 3.56	2.12 ${\pm }$ 0.160	2.13 ${\pm }$ 0.143
**Right kidney**			
DICE	0.802 ${\pm }$ 0.107	0.721 ${\pm }$ 0.265	0.841 ${\pm }$ 0.078
JACCARD	0.680 ${\pm }$ 0.132	0.608 ${\pm }$ 0.239	0.732 ${\pm }$ 0.109
VS	0.832 ${\pm }$ 0.067	0.818 ${\pm }$ 0.226	0.893 ${\pm }$ 0.073
HD	12.0 ${\pm }$ 11.5	23.8 ${\pm }$ 18.6	12.3 ${\pm }$ 6.58
TPR	0.725 ${\pm }$ 0.073	0.797 ${\pm }$ 0.293	0.839 ${\pm }$ 0.163
TNR	0.999 ${\pm }$ 0.004	0.999 ${\pm }$ 0.001	0.999 ${\pm }$ 0.0001
Time (s)	30.5 ${\pm }$ 4.09	2.06 ${\pm }$ 0.093	2.05 ${\pm }$ 0.069
**Spleen**			
DICE	0.890 ${\pm }$ 0.034	0.886 ${\pm }$ 0.065	0.853 ${\pm }$ 0.224
JACCARD	0.804 ${\pm }$ 0.051	0.801 ${\pm }$ 0.098	0.785 ${\pm }$ 0.239
VS	0.923 ${\pm }$ 0.031	0.922 ${\pm }$ 0.069	0.896 ${\pm }$ 0.236
HD	6.57 ${\pm }$ 5.08	14.2 ${\pm }$ 7.30	40.0 ${\pm }$ 52.4
TPR	0.828 ${\pm }$ 0.025	0.892 ${\pm }$ 0.130	0.922 ${\pm }$ 0.066
TNR	0.999 ${\pm }$ 0.001	0.999 ${\pm }$ 0.0001	0.999 ${\pm }$ 0.0001
Time (s)	29.7 ${\pm }$ 4.09	2.06 ${\pm }$ 0.010	2.08 ${\pm }$ 0.115
**Aorta**			
DICE	0.731 ${\pm }$ 0.113	0.721 ${\pm }$ 0.169	0.847 ${\pm }$ 0.124
JACCARD	0.585 ${\pm }$ 0.121	0.587 ${\pm }$ 0.198	0.750 ${\pm }$ 0.160
VS	0.769 ${\pm }$ 0.077	0.879 ${\pm }$ 0.102	0.936 ${\pm }$ 0.067
HD	6.71 ${\pm }$ 9.20	38.4 ${\pm }$ 24.7	13.7 ${\pm }$ 14.9
TPR	0.646 ${\pm }$ 0.060	0.696 ${\pm }$ 0.200	0.821 ${\pm }$ 0.162
TNR	0.999 ${\pm }$ 0.003	0.999 ${\pm }$ 0.001	0.999 ${\pm }$ 0.0001
Time	30.9 ${\pm }$ 4.71	2.04 ${\pm }$ 0.085	2.05 ${\pm }$ 0.092
**Liver**			
DICE	0.928 ${\pm }$ 0.027	0.930 ${\pm }$ 0.032	0.941 ${\pm }$ 0.028
JACCARD	0.867 ${\pm }$ 0.045	0.870 ${\pm }$ 0.055	0.890 ${\pm }$ 0.049
VS	0.966 ${\pm }$ 0.017	0.970 ${\pm }$ 0.034	0.975 ${\pm }$ 0.034
HD	13.8 ${\pm }$ 8.81	24.4 ${\pm }$ 15.2	11.8 ${\pm }$ 7.96
TPR	0.911 ${\pm }$ 0.015	0.947 ${\pm }$ 0.031	0.949 ${\pm }$ 0.037
TNR	0.996 ${\pm }$ 0.004	0.999 ${\pm }$ 0.0001	0.999 ${\pm }$ 0.0001
Time (s)	31.0 ${\pm }$ 3.29	2.04 ${\pm }$ 0.091	2.03 ${\pm }$ 0.092

**Table 7:** Recently published segmentation experiments on cross-sectional images of solid organs or lesions.

	Region	Data	DICE	JACCARD
**Aortic**				
Lalys et al.^30^	Whole Aneurysm	92 preop CT scans	0.86 ${\pm }$ 0.06	0.82 ${\pm }$ 0.07
		15 postop CT scans	0.87 ${\pm }$ 0.03	0.83 ${\pm }$ 0.04
Lareyre et al.^32^	Lumen	40 preop CT scans	0.93 ${\pm }$ 0.04	0.87 ${\pm }$ 0.07
	Thrombus		0.88 ${\pm }$ 0.12	0.80 ${\pm }$ 0.15
Lopez et al.^35^	Thrombus	12 preop CT scans	0.84 ${\pm }$ 0.07	0.73 ${\pm }$ 0.10
	Thrombus	16 postop CT scans	0.89 ${\pm }$ 0.04	0.81 ${\pm }$ 0.07
Lu et al.^36^	Whole Aneurysm	57 preop CT scans	0.89 ${\pm }$ 0.05	–
Caradu et al.^38^	Whole Aneurysm	100 preop CT scans	0.95 ${\pm }$ 0.01	0.91 ${\pm }$ 0.02
Adam et al.^39^	Thoracic Aorta, Whole Aneurysm, Iliac Sections	22 postop CT scans	0.93	–
Proposed	Thrombus	10 postop CT scans	0.91 ${\pm }$ 0.05	0.84 ${\pm }$ 0.09
	Stent and Lumen		0.94 ${\pm }$ 0.03	0.88 ${\pm }$ 0.05
	Whole Aneurysm		0.93 ${\pm }$ 0.04	0.88 ${\pm }$ 0.07
**BraTS**				
Jiang et al.^40^	Whole tumour	125 MRI scans	0.89	–
Zhao et al.^41^			0.88	–
Ali et al.^42^			0.91	–
Proposed		40 MRI scans	0.83 ${\pm }$ 0.13	0.73 ${\pm }$ 0.17
**Abdomen**				
Gibson et al.^43^	Left Kidney	90 CT scans	0.93	–
	Spleen		0.95	–
	Liver		0.95	–
Cai et al.^44^	Spleen	10 CT scans	0.96 ${\pm }$ 0.01	–
	Liver	32 CT scans	0.94 ${\pm }$ 0.03	–
Weston et al.^45^	Left and right kidneys	18 CT scans	0.93	–
	Spleen		0.93	–
	Aorta		0.88	–
	Liver		0.95	–
Proposed	Left kidney	10 CT scans	0.89 ${\pm }$ 0.08	0.82 ${\pm }$ 0.11
	Right kidney		0.84 ${\pm }$ 0.08	0.73 ${\pm }$ 0.11
	Spleen		0.85 ${\pm }$ 0.22	0.79 ${\pm }$ 0.24
	Aorta		0.85 ${\pm }$ 0.12	0.75 ${\pm }$ 0.16
	Liver		0.94 ${\pm }$ 0.02	0.89 ${\pm }$ 0.05

## Supplementary Information

Supplementary Information.

## Data Availability

The BraTS dataset is publically available from https://www.med.upenn.edu/cbica/brats2020/data.html. The Abdomen datset is publically available from https://www.synapse.org/#Synapse:syn3193805/wiki/217789. The aortic data that support the findings of this study are available from Royal Liverpool and Broadgreen University Hospitals but restrictions apply to the availability of these data, which were used under license for the current study, and so are not publicly available.

## References

[1] Mumford DB, Shah J (1989). Optimal approximations by piecewise smooth functions and associated variational problems. Commun. Pure Appl. Math..

[2] Chan TF, Vese LA (2001). Active contours without edges. IEEE Trans. Image Process..

[3] Cai X, Chan R, Zeng T (2013). A two-stage image segmentation method using a convex variant of the mumford-shah model and thresholding. SIAM J. Imag. Sci..

[4] Ronneberger, O., Fischer, P. & Brox, T. U-net: Convolutional networks for biomedical image segmentation. In *International Conference on Medical Image Computing and Computer-Assisted Intervention*, 234–241 (Springer, 2015).

[5] Chen L-C, Papandreou G, Kokkinos I, Murphy K, Yuille AL (2017). Deeplab: Semantic image segmentation with deep convolutional nets, atrous convolution, and fully connected crfs. IEEE Trans. Pattern Anal. Mach. Intell..

[6] Chen, L.-C., Zhu, Y., Papandreou, G., Schroff, F. & Adam, H. Encoder-decoder with atrous separable convolution for semantic image segmentation. In *Proceedings of the European Conference on Computer Vision (ECCV),* 801–818 (2018).

[7] Tang M, Valipour S, Zhang Z, Cobzas D, Jagersand M (2017). A deep level set method for image segmentation. Deep Learning in Medical Image Analysis and Multimodal Learning for Clinical Decision Support.

[8] Chen, X. et al. Learning active contour models for medical image segmentation. In *Proceedings of the IEEE/CVF Conference on Computer Vision and Pattern Recognition,* 11632–11640 (2019).

[9] Burrows, L., Chen, K. & Torella, F. On new convolutional neural network based algorithms for selective segmentation of images. In *Annual Conference on Medical Image Understanding and Analysis*, 93–104 (Springer, 2020).

[10] Burrows L, Guo W, Chen K, Torella F (2021). Reproducible kernel hilbert space based global and local image segmentation. Inverse Probl. Imaging.

[11] Shaikh U (2015). Changes in aortic volumes following endovascular sealing of abdominal aortic aneurysms with the nellix endoprosthesis. J. Endovasc. Ther..

[12] Yafawi A (2020). Aneurysm growth after endovascular sealing of abdominal aortic aneurysms (evas) with the nellix endoprosthesis. Eur. J. Vasc. Endovasc. Surg..

[13] Yafawi A (2019). Stent frame movement following endovascular aneurysm sealing in the abdominal aorta. J. Endovasc. Ther..

[14] Yushkevich PA (2006). User-guided 3d active contour segmentation of anatomical structures: Significantly improved efficiency and reliability. Neuroimage.

[15] Menze BH (2014). The multimodal brain tumor image segmentation benchmark (brats). IEEE Trans. Med. Imaging.

[16] Bakas S (2017). Advancing the cancer genome atlas glioma mri collections with expert segmentation labels and radiomic features. Sci. Data.

[17] Bakas, S. *et al*. Identifying the best machine learning algorithms for brain tumor segmentation, progression assessment, and overall survival prediction in the brats challenge. arXiv:1811.02629 (arXiv preprint) (2018).

[18] Landman, B. *et al.* Segmentation outside the cranial vault challenge. https://repo-prod.prod.sagebase.org/repo/v1/doi/locate?id=syn3193805&type=ENTITY. 10.7303/SYN3193805 (2015).

[19] Theljani, A. & Chen, K. An unsupervised deep learning method for diffeomorphic mono-and multi-modal image registration. In *Annual Conference on Medical Image Understanding and Analysis*, 317–326 (Springer, 2019).

[20] Bland JM, Altman D (1986). Statistical methods for assessing agreement between two methods of clinical measurement. Lancet.

[21] Soltaninejad M (2018). Supervised learning based multimodal mri brain tumour segmentation using texture features from supervoxels. Comput. Methods Programs Biomed..

[22] Chen C (2020). Deep learning for cardiac image segmentation: A review. Front. Cardiovasc. Med..

[23] Hesamian MH, Jia W, He X, Kennedy P (2019). Deep learning techniques for medical image segmentation: Achievements and challenges. J. Digit. Imaging.

[24] Wang G (2018). Interactive medical image segmentation using deep learning with image-specific fine tuning. IEEE Trans. Med. Imaging.

[25] Wang G (2018). Deepigeos: A deep interactive geodesic framework for medical image segmentation. IEEE Trans. Pattern Anal. Mach. Intell..

[26] Loncaric, S., Subasic, M. & Sorantin, E. 3-d deformable model for aortic aneurysm segmentation from ct images. In *Proceedings of the 22nd Annual International Conference of the IEEE Engineering in Medicine and Biology Society (Cat. No. 00CH37143)*, vol. 1, 398–401 (IEEE, 2000).

[27] Subasic, M., Loncaric, S. & Sorantin, E. Region-based deformable model for aortic wall segmentation. In *3rd International Symposium on Image and Signal Processing and Analysis, 2003. ISPA 2003. Proceedings of the*, vol. 2, 731–735 (IEEE, 2003).

[28] Subašić M, Lončarić S, Sorantin E (2005). Model-based quantitative aaa image analysis using a priori knowledge. Comput. Methods Programs Biomed..

[29] Zohios C, Kossioris G, Papaharilaou Y (2012). Geometrical methods for level set based abdominal aortic aneurysm thrombus and outer wall 2d image segmentation. Comput. Methods Programs Biomed..

[30] Lalys F, Yan V, Kaladji A, Lucas A, Esneault S (2017). Generic thrombus segmentation from pre-and post-operative cta. Int. J. Comput. Assist. Radiol. Surg..

[31] Kass M, Witkin A, Terzopoulos D (1988). Snakes: Active contour models. Int. J. Comput. Vis..

[32] Lareyre F (2019). A fully automated pipeline for mining abdominal aortic aneurysm using image segmentation. Sci. Rep..

[33] López-Linares K (2018). Fully automatic detection and segmentation of abdominal aortic thrombus in post-operative cta images using deep convolutional neural networks. Med. Image Anal..

[34] Xie, S. & Tu, Z. Holistically-nested edge detection. In *Proceedings of the IEEE International Conference on Computer Vision*, 1395–1403 (2015).

[35] López-Linares, K., García, I., García-Familiar, A., Macía, I. & Ballester, M. A. G. 3d convolutional neural network for abdominal aortic aneurysm segmentation. arXiv:1903.00879 (arXiv preprint) (2019). 10.1007/978-3-030-33327-0_20 PMC8188890 34113925

[36] Lu, J.-T. *et al*. Deepaaa: clinically applicable and generalizable detection of abdominal aortic aneurysm using deep learning. In *International Conference on Medical Image Computing and Computer-Assisted Intervention*, 723–731 (Springer, 2019).

[37] Milletari, F., Navab, N. & Ahmadi, S.-A. V-net: Fully convolutional neural networks for volumetric medical image segmentation. In *2016 Fourth International Conference on 3D Vision (3DV)*, 565–571 (IEEE, 2016).

[38] Caradu C, Spampinato B, Vrancianu AM, Bérard X, Ducasse E (2021). Fully automatic volume segmentation of infrarenal abdominal aortic aneurysm computed tomography images with deep learning approaches versus physician controlled manual segmentation. J. Vasc. Surg..

[39] Adam C (2021). Pre-surgical and post-surgical aortic aneurysm maximum diameter measurement: Full automation by artificial intelligence. Eur. J. Vasc. Endovasc. Surg..

[40] Jiang, Z., Ding, C., Liu, M. & Tao, D. Two-stage cascaded u-net: 1st place solution to brats challenge 2019 segmentation task. In *International MICCAI Brainlesion Workshop*, 231–241 (Springer, 2019).

[41] Zhao, Y.-X., Zhang, Y.-M. & Liu, C.-L. Bag of tricks for 3d mri brain tumor segmentation. In *International MICCAI Brainlesion Workshop*, 210–220 (Springer, 2019).

[42] Ali M, Gilani SO, Waris A, Zafar K, Jamil M (2020). Brain tumour image segmentation using deep networks. IEEE Access.

[43] Gibson E (2018). Automatic multi-organ segmentation on abdominal ct with dense v-networks. IEEE Trans. Med. Imaging.

[44] Cai, J. *et al*. End-to-end adversarial shape learning for abdomen organ deep segmentation. In *International Workshop on Machine Learning in Medical Imaging*, 124–132 (Springer, 2019).

[45] Weston AD (2020). Complete abdomen and pelvis segmentation using u-net variant architecture. Med. Phys..

