# Supplementary material for: Evaluation of a hybrid pipeline for automated segmentation of solid lesions based on mathematical algorithms and deep learning
Source: Sci Rep. 2022 Aug 20;12:14216. doi: 10.1038/s41598-022-18173-0 (PMC9392778; doi:10.1038/s41598-022-18173-0)
Supplement: Supplementary file 1 — Supplementary Information. [file 41598_2022_18173_MOESM1_ESM.pdf]

Evaluation of a Hybrid Pipeline for Automated Segmentation of Solid Lesions based on Mathematical Algorithms and Deep Learning

Liam Burrows[1,*], Ke Chen[1,*], Weihong Guo[2], Martin Hossack[3], Richard G McWilliams[4], and Francesco Torella[3]

[1]Centre for Mathematical Imaging Techniques and Department of Mathematical Sciences University of Liverpool, Liverpool, L69 7ZL, United Kingdom
[2]Department of Mathematics, Applied Mathematics and Statistics, Case Western Reserve University, Cleveland, OH 44106, USA
[3]Liverpool Vascular & Endovascular Service, Liverpool University Hospitals NHS Foundation Trust, Liverpool, United Kingdom
[4]Department of Radiology, Liverpool University Hospitals, Liverpool, United Kingdom
[*]liam.burrows@liv.ac.uk, k.chen@liv.ac.uk

Appendix

Implementation Details

Here we outline some practical aspects of the implementation, and provide some more detail to the deep learning part of the method. Our proposed segmentation network for the hybrid approach uses an estimated segmentation provided by the variational method We use a DICE loss function, extending it to incorporate the unlabelled part. Let us denote $u(i)$ as the segmentation output of our network, $v(i)$ the segmentation output of the variational method in the previous step, and $GT(i)$ the ground truth segmentation for the ith training image. If we have L labelled images and U unlabelled images, for a total of $N = L+U$ images, the loss function we use is given as:

$$\mathscr{L}(\Theta) = \sum_{i=1}^{L}(1-DICE(GT(i),u(i))) + \xi \sum_{i=1}^{U}(1-DICE(v(i),u(i))), \tag{1}$$

where $\xi > 0$ is a parameter and DICE is the DICE similarity coefficient. Incorporating a mix of labelled and unlabelled data in this fashion is known as a semi-supervised approach for deep learning. The architecture of the network is given in Figure 1.

The standard model is similar but does not make use of the output from the variational model $v(i)$, and therefore does not make use of unlabelled images. Therefore, the loss function is given in (1) with $\xi = 0$, and the architecture is the same as that shown in for the hybrid approach in Figure 1, except the input is only the image z rather than the image and $v(i)$.

As both approaches use a very similar network, the training times for both are similar. For the aortic data, as the hybrid approach had unlabelled data available, the training took roughly 18 hours, whereas the standard approach took closer to 14 hours. In the case of the BraTS dataset, both approaches took 12 hours to train, and the Abdomen dataset took roughly 8 hours per organ to train.

We use an Adam optimiser with a learning rate of 0.01, and all implementation is done in Tensorflow 2.3.

Further experiments

We ran some additional experiments involving training the hybrid approach with a decreasing number of images in the training set, in order to examine the difference between the hybrid approach with reduced data and the standard approach with full data. A table of results of the hybrid approach with varying amounts of training data can be found in Table 1. Each row displays a segmented object and the number of datasets in the training set for that particular case. For all but the spleen, at least one of the hybrid networks trained with less data outperforms the standard network with full data, though there are clear limitations (for example, aortic data with only 10 training datasets).

Included in Table 1 is results from the aortic data trained with 30 images. The original experiments on the aortic data were semi-supervised as 20 unlabelled images were available, however the rows showing the aortic data trained with 30 images in Table 1 show the hybrid network trained without the 20 unlabelled images. This shows the difference in performance between a supervised and semi-supervised model, where the supervised model gives a mean DICE score of 0.889 for the thrombus, whereas incorporating the unlabelled component brings the mean score to 0.909.

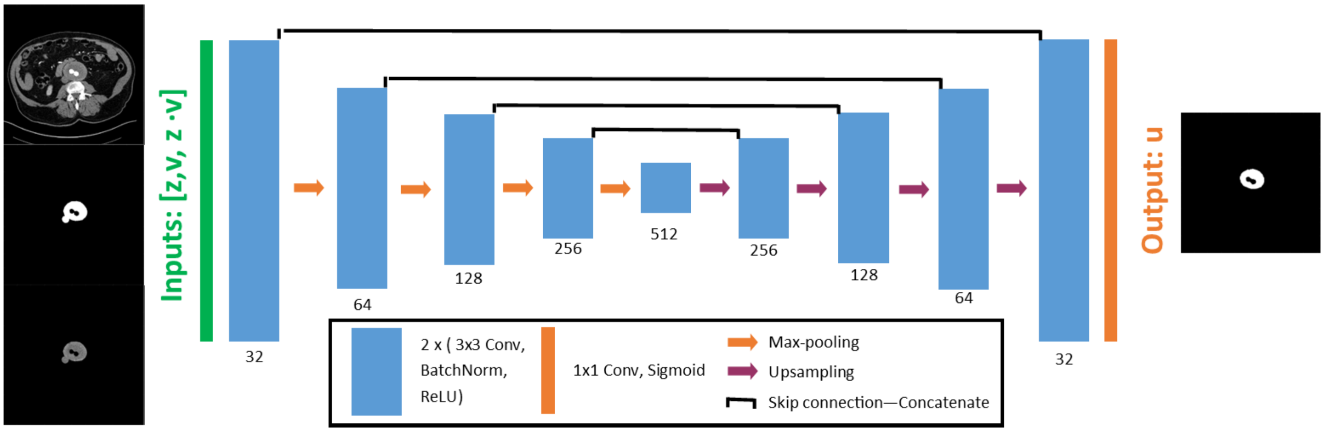

Figure 1. The network architecture of our CNN.

We also trained the hybrid approach using lighter network architectures to compare the effects. The two networks, which we name "LightNet" (LNet) and "SimplifiedNet" (SNet) are similar to the original proposed networks except: LNet has less filters in each convolutional layer, and SNet has one less max-pooling/upsampling step. Both of these networks can be found illustrated in Figure 2, and tables from training these networks on the datasets can be found in Table 2

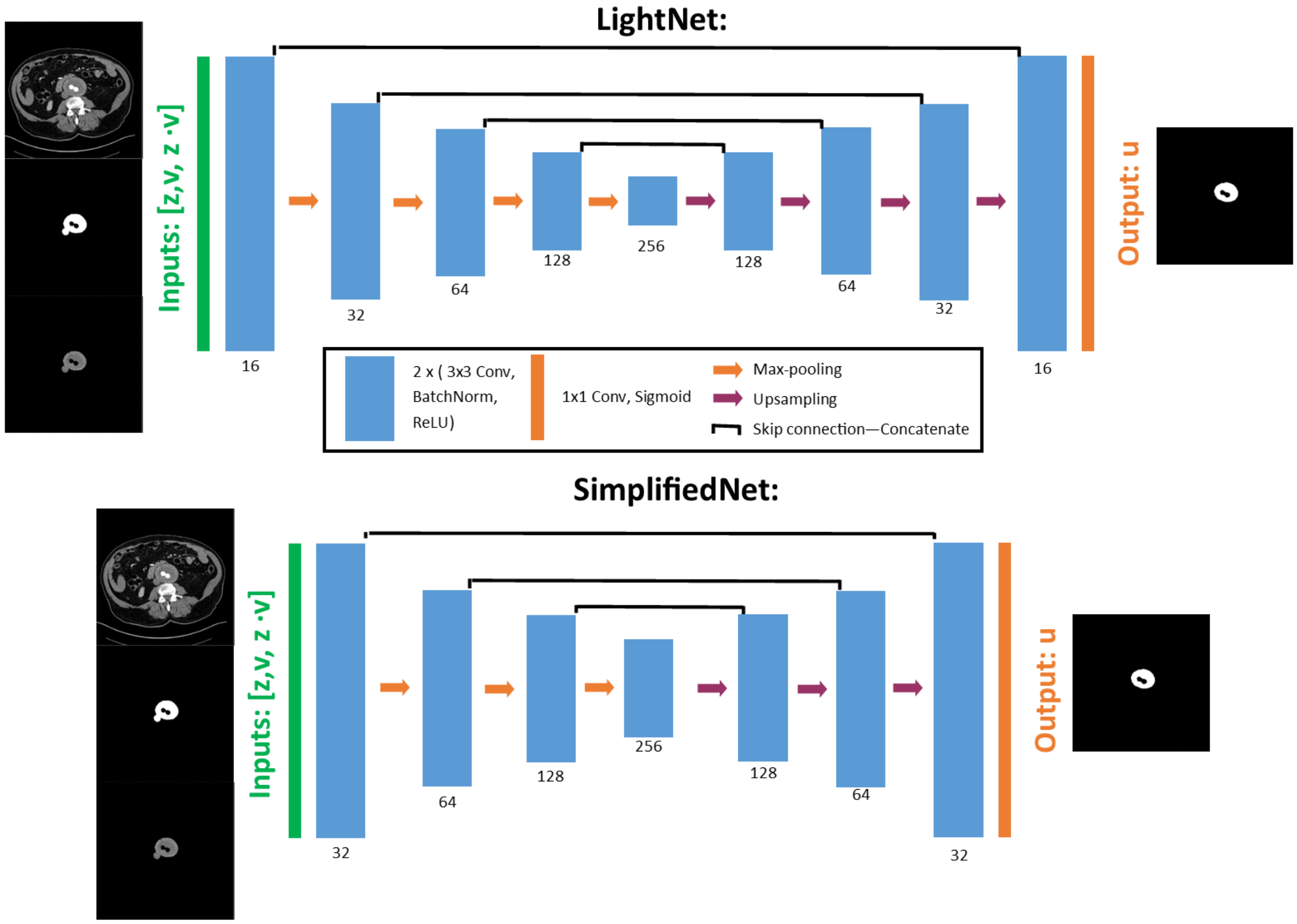

Figure 2. Lighter network architectures.

	DICE	JAC	VS	HD	TPR	TNR
**Thrombus 30**	0.889±0.093	0.810±0.138	0.940±0.088	45.9±36.8	0.927±0.046	0.998±0.003
**Thrombus 20**	0.877±0.133	0.781±0.168	0.899±0.130	78.9±55.6	0.915±0.052	0.997±0.003
**Thrombus 15**	0.731±0.154	0.595±0.171	0.818±0.183	34.7±13.8	0.896±0.049	0.993±0.006
**Thrombus 10**	0.695±0.166	0.554±0.180	0.817±0.168	75.9±35.3	0.822±0.087	0.993±0.006
**S & L 30**	0.950±0.024	0.905±0.043	0.982±0.010	49.6±62.0	0.954±0.028	0.998±0.001
**S & L 20**	0.927±0.066	0.864±0.090	0.969±0.090	105±26.4	0.943±0.032	0.999±0.0008
**S & L 15**	0.896±0.070	0.817±0.104	0.930±0.070	56.2±61.7	0.848±0.110	0.999±0.0001
**S & L 10**	0.801±0.059	0.672±0.081	0.923±0.053	67.7±50.2	0.809±0.061	0.999±0.0007
**Whole 30**	0.921±0.076	0.862±0.119	0.954±0.073	62.4±47.9	0.953±0.034	0.998±0.003
**Whole 20**	0.903±0.107	0.831±0.149	0.944±0.099	95.4±61.0	0.926±0.060	0.997±0.003
**Whole 15**	0.778±0.133	0.652±0.163	0.849±0.156	59.5±55.2	0.915±0.042	0.993±0.006
**Whole 10**	0.757±0.137	0.625±0.163	0.837±0.150	115±29.8	0.889±0.069	0.993±0.007
**BraTS 80**	0.819±0.127	0.693±0.164	0.859±0.132	38.2±19.3	0.875±0.146	0.996±0.007
**BraTS 60**	0.761±0.200	0.651±0.236	0.787±0.208	39.4±25.5	0.888±0.160	0.986±0.026
**L Kidney 15**	0.874±0.063	0.781±0.092	0.940±0.078	9.42±5.35	0.851±0.116	0.999±0.0006
**L Kidney 10**	0.867±0.068	0.771±0.101	0.910±0.054	9.44±7.90	0.903±0.128	0.998±0.001
**R Kidney 15**	0.821±0.143	0.696±0.182	0.880±0.123	18.3±18.1	0.781±0.162	0.996±0.004
**R Kidney 10**	0.807±0.202	0.76±0.220	0.866±0.106	21.7±15.0	0.771±0.201	0.996±0.005
**Spleen 15**	0.844±0.223	0.771±0.238	0.887±0.234	25.1±37.9	0.924±0.069	0.991±0.025
**Spleen 10**	0.841±0.222	0.766±0.235	0.886±0.234	16.4±23.1	0.930±0.057	0.990±0.026
**Aorta 15**	0.840±0.101	0.735±0.141	0.907±0.090	14.7±16.4	0.825±0.164	0.999±0.0004
**Aorta 10**	0.823±0.096	0.700±0.135	0.880±0.095	17.5±14.1	0.817±0.158	0.999±0.0001
**Liver 15**	0.935±0.015	0.877±0.027	0.972±0.019	17.2±23.1	0.943±0.030	0.995±0.004
**Liver 10**	0.922±0.023	0.856±0.039	0.968±0.027	15.3±7.75	0.918±0.041	0.994±0.004

Table 1. Organ segmentation for all the organs/lesions using the proposed hybrid network with reduced training data.

		DICE	JAC	VS	HD	TPR	TNR
**Thrombus**	**LNet**	0.807±0.127	0.692±0.157	0.882±0.147	30.2±16.0	0.902±0.066	0.996±0.003
	**SNet**	0.817±0.149	0.711±0.188	0.882±0.163	26.7±15.8	0.914±0.066	0.996±0.004
**S & L**	**LNet**	0.904±0.037	0.827±0.060	0.949±0.029	59.0±58.1	0.868±0.060	0.999±0.0001
	**SNet**	0.935±0.045	0.881±0.074	0.967±0.035	56.3±60.3	0.931±0.077	0.999±0.0001
**Whole**	**LNet**	0.853±0.102	0.755±0.140	0.905±0.114	55.2±58.1	0.922±0.059	0.996±0.004
	**SNet**	0.858±0.120	0.768±0.166	0.903±0.131	54.0±57.4	0.937±0.061	0.996±0.004
**BraTS**	**LNet**	0.693±0.264	0.587±0.287	0.714±0.275	51.3±24.2	0.889±0.176	0.974±0.040
	**SNet**	0.824±0.137	0.721±0.172	0.856±0.144	23.3±15.6	0.891±0.159	0.994±0.008
**L Kidney**	**LNet**	0.872±0.093	0.782±0.130	0.928±0.093	7.17±3.86	0.880±0.154	0.999±0.001
	**SNet**	0.887±0.065	0.802±0.100	0.944±0.070	11.5±10.7	0.865±0.115	0.999±0.0004
**R Kidney**	**LNet**	0.851±0.107	0.753±0.142	0.953±0.052	13.9±18.2	0.848±0.126	0.998±0.001
	**SNet**	0.846±0.061	0.738±0.090	0.922±0.054	18.0±19.8	0.893±0.101	0.997±0.002
**Spleen**	**LNet**	0.845±0.210	0.769±0.228	0.892±0.223	16.0±23.2	0.903±0.078	0.992±0.020
	**SNet**	0.847±0.166	0.760±0.194	0.897±0.177	15.1±21.2	0.877±0.077	0.995±0.011
**Aorta**	**LNet**	0.859±0.093	0.762±0.130	0.934±0.083	13.7±15.1	0.835±0.147	0.999±0.0002
	**SNet**	0.849±0.103	0.749±0.140	0.919±0.091	13.9±14.0	0.838±0.163	0.999±0.0001
**Liver**	**LNet**	0.942±0.019	0.890±0.032	0.980±0.024	11.1±3.74	0.932±0.038	0.996±0.004
	**SNet**	0.946±0.019	0.897±0.033	0.983±0.024	14.8±8.55	0.940±0.038	0.996±0.004

Table 2. Organ segmentation for all the organs/lesions from networks similar to the original proposed hybrid network, but with reduced weight.
